# Items

**Published:** 1910-01

**Authors:** 


### ITEMS.

BATTLE & Co., of St. Louis, have just issued No. 11 of their series of charts on dislocations. This series forms a most valuable and interesting addition to any physician's library. They will be sent free of charge on application, and back numbers will also be supplied. It is important to write Battle & Co., for missing numbers before the supply is exhausted.

THE Physician's Year Book for 1910, made its appearance about December 15, 1909. It is a useful daily memorandum suitable for the office desk and contains blank spaces for every day in the year. Those who have used it heretofore will be glad to possess it again. It is published by the M. J. Breitenbach Co., 53 Warren Street, New York, and will be sent post free on application.

TARRANT'S Seltzer Aperient maintains the standard it established years ago, as a most valuable remedy in gouty and rheumatic affections in which an alkaline laxative is needed. Its effervescent properties make it agreeable to most persons, while its efficiency as an eliminator gives it a strong claim to high place among the therapeutic sources of physicians who have tested it in appropriate cases.

THE Antikamnia Chemical Company, Saint Louis, has issued its annual calendar. This one, 1910, is a reproduction in color on the obverse of "Beatrice." On the reverse is the tablet calendar together with some indications for the use of the Antikamnia tablets. The picture is an artistic reproduction of the celebrated painting. A duplicate of the calendar will be sent by the company on receipt of six cents in stamps.

CALOX, the oxygen tooth powder, made by McKessen & Robbins, should not be overlooked at this season of the year. It not only cleans the teeth, but it cleanses the mouth, frees it of germs, and leaves a wholesome taste in its trail. Of all the various tooth powders on the market, Calox should be given first place by physicians and dentists, who certainly know the importance of preserving the teeth as well as keeping them clean. Calox may well be included among the useful holiday gifts.

